# Online Interest in Elf Bar in the United States: Google Health Trends Analysis

**DOI:** 10.2196/50343

**Published:** 2024-11-05

**Authors:** Akshaya Srikanth Bhagavathula, Page D Dobbs

**Affiliations:** 1 Department of Public Health North Dakota State University Fargo, ND United States; 2 Department of Health, Human Performance and Recreation University of Arkansas Fayetteville, AR United States

**Keywords:** e-cigarettes, Elf Bar, JUUL, tobacco, Google Trends, Google Health Trends

## Abstract

**Background:**

Despite the popularity of JUUL e-cigarettes, other brands (eg, Elf Bar) may be gaining digital attention.

**Objective:**

This study compared Google searches for Elf Bar and JUUL from 2022 to 2023 using Google Health Trends Application Programming Interface data.

**Methods:**

Using an infodemiology approach, we examined weekly trends in Google searches (per 10 million) for “Elf Bar” and “JUUL” at the US national and state levels from January 1, 2022, to December 31, 2023. Joinpoint regression was used to assess statistically significant trends in the search probabilities for “Elf Bar” and “JUUL” during the study period.

**Results:**

Elf Bar had less online interest than JUUL at the beginning of 2022. When the US Food and Drug Administration denied JUUL marketing authority on June 23, 2022, JUUL searches peaked at 2609.3 × 10^7^ and fell to 83.9 × 10^7^ on September 3, 2023. Elf Bar searches surpassed JUUL on July 10, 2022, and steadily increased, reaching 523.2 × 10^7^ on December 4, 2022. Overall, Elf Bar’s weekly search probability increased by 1.6% (95% CI 1.5%-1.7%; *P*=.05) from January 2022 to December 2023, with the greatest increase between May 29 and June 19, 2022 (87.7%, 95% CI 35.9%-123.9%; *P*=.001). Elf Bar searches increased after JUUL’s suspension in Pennsylvania (1010%), Minnesota (872.5%), Connecticut (803.5%), New York (738.1%), and New Jersey (702.9%).

**Conclusions:**

Increasing trends in Google searches for Elf Bar indicate that there was a growing online interest in this brand in the United States in 2022.

## Introduction

Defined by 4 distinct “generations,” the first e-cigarette products resembled traditional cigarettes and were referred to as “cig-a-likes” [1]. These devices evolved into vape pens (second generation) and modifiable tank-style systems (third generation). The current (fourth) generation of e-cigarette products now include cartridge-based and disposable devices. Cartridge-based devices include a prefilled “pod” that contains nicotine and flavors and can be discarded after use, and disposable devices are advertised to include a number of “puffs” (eg, 1500), after which they can be disposed [1]. Although youth and young adults’ use of e-cigarettes was increasing from 2011 to 2020 [2], it peaked in 2019, when the fourth-generation flash drive–shaped product, JUUL, controlled the e-cigarette market [3-5]. Although the use of e-cigarettes has declined since 2019, they continue to be popular among young audiences, with 2.13 million middle and high school students reporting current use in the United States in 2023 [6].

In January 2020, the Food and Drug Administration (FDA) announced that a sales restriction of cartridge-based e-cigarette flavors (other than mint and tobacco) would go into effect later that month [7]. This policy was suggested to decrease the popularity of cartridge-based e-cigarettes such as JUUL. JUUL Labs Inc has now settled in court regarding cases where they were accused of marketing their products to those younger than 21 years, the legal sales age for tobacco products [8]. Although this policy was posited to reduce use among youth and young adults, researchers have speculated that the FDA’s cartridge-based flavor restriction policy created a loophole for disposable devices, such as Puff Bar, that gained online attention after this policy was enacted [9,10].

On July 20, 2020, the FDA issued warning letters to 10 companies, including Puff Bar and other disposable e-cigarette brands, that they had not received the appropriate premarket authority to be marketed and sold in the United States [11]. After a short period of suspended sales, Puff Bar began selling their products again in March 2021, claiming that they used synthetic nicotine, which was not authorized for regulation by the FDA [12]; the FDA’s authority over nicotine products is defined in the Tobacco Control Act as products that were made, contained, or derived from tobacco [13]. Thus, on March 11, 2022, President Joe Biden closed this regulatory gap by signing a Congress-approved amendment (Docket No. FDA-2022-N-3261) that expanded the tobacco product definition to “products that contain nicotine from any source,” granting the FDA authority over synthetic nicotine products [12].

From 2021 to 2023, other brands of disposable e-cigarette devices, including Elf Bar, began to gain popularity in Great Britain and the United States, with 56.7% of US youths who reported current use of e-cigarettes indicating that they used Elf Bar most often in 2023 (compared with 15.5% for JUUL) [14-17]. Research indicates that discussion about Elf Bar and other emerging e-cigarette brands was increasing on Twitter in 2021, whereas discussion about JUUL was decreasing [18]. During this time, research suggests that disposable, nicotine salt–based e-cigarette products became the most popular e-cigarette devices on the market [15]. However, no research has explored the popularity of Elf Bar on a more widely available search engine, such as Google. Google Trends (GT) is a useful tool for measuring popularity (defined as online interest) of a wide range of topics including football players, disease prioritization, and the harms of drug use [19-21]. It has been used in prior tobacco-related research, to examine the online popularity of JUUL and Puff Bar [10], but it has not been used since the FDA’s expansion of the tobacco product definition to determine the online popularity of other e-cigarette products, such as Elf Bar.

June 2022, the FDA announced the prohibition of JUUL sales in the US market [22], an order that was immediately appealed. Thus, although JUUL remained on the market, the popularity of this product continued to decline while newer disposable products that included flavors saw increases in use [9,10,23]. Although data suggest that there were shifts in e-cigarette product and brand use after the COVID-19 pandemic [15], and research has noted the popularity of Puff Bar [9,24,25], there remains a gap in literature about the online interest in other brands, particularly following the FDA’s order to suspend Puff Bar sales. It is possible that regulatory announcements could have created changes in online interest of e-cigarette products. With growing regulations that focus on particular brand or product characterizations (eg, cartridge-based systems and synthetic nicotine-containing products), there could be growing online popularity for other e-cigarette brands, such as Elf Bar, a brand that has been noted for its recent popularity in the United Kingdom and United States [16,17]. The purpose of this study is to explore the online interest in Elf Bar in the United States, compared with JUUL from 2022 to 2023 using Google Health Trends Application Programming Interface (GHT-API) data.

## Methods

### GHT-API Data

GT is a popular data source that is widely used in public health research [26]. Given recent concerns about the data quality and accuracy of GT [27-29], some researchers have begun to use GHT-API, which provides raw probabilities of a short search session with no restrictions on the search volume index [30,31]. To collect and use the GHT-API data, researchers must have a valid API key supplied by Google. A detailed GHT-API technique and data features have already been published elsewhere [32,33]. Briefly, Google maintains a random sample of all Google search queries that can be obtained by researchers using the API. The GHT-API provides researchers with the relative share of all Google searches made by individuals within a specific geographic area and time range for given search terms. The API returns a scale proportion equal to the number of searches for specific search terms as a proportion of total searches multiplied by a constant of 10 million. Compared with traditional GT metrics, GHT-API facilitates more granular and unbiased analyses of temporal and spatial search trends pertinent to public health topics. Further research leveraging this promising data source may elucidate novel insights into online health information–seeking behaviors.

To identify Google search terms, we examined GT search queries to identify the most popular queries related to “Elf Bar” and “JUUL” from 2022 to 2023. Given JUUL’s prior popularity [10], we included the term JUUL as a reference that would help interpret the popularity of Elf Bar as an emerging e-cigarette product. The study considered search queries labeled “Breakout” (indicates that the Google search term has grown by more than 5000%) [34]. Following these techniques and using a valid GHT-API key, we collected the data for the search terms “Elf Bar” and “JUUL” in the United States from January 1, 2022, through December 31, 2023, a time at which Elf Bar was noted for its popularity among US youth [17]. Like previous studies [30-33], we collected multiple samples (using the same dates) of weekly and monthly data searches for “Elf Bar” and “JUUL” within the United States and averaged them to obtain better estimates of their true values. We conducted weekly and monthly searches at the state level for all 50 states between January 1 and October 16, 2022; however, 8 states (Alaska, Delaware, Montana, North Dakota, Rhode Island, South Dakota, Vermont, and Wyoming) and the District of Columbia were excluded because of insufficient data or suppression of the API by the GHT-API. These areas were not considered in the state-level analysis.

### Statistical Analysis

To obtain GHT-API data, Python 3.11.0 (Python Software Foundation) was used. Excel (Microsoft Corp) was used to clean data, and the *geofacet* package for R version 4.2.1 (R Foundation for Statistical Computing) was used to create data visualizations. The coefficient of variance (COV) method was used to calculate the variability of data between samples. The COV can be calculated as the ratio of the SD to the mean:



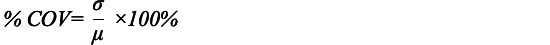



where *μ* is the mean probability of searches and *σ* is the SD.

The COV measures how consistent the values of each sample are from the representative mean of the data samples. The lower the percentage COV value, the greater the homogeneity of the values in the data set. A total of 5 samples were collected with COV that were found to be less than 5% between the samples. Therefore, we used the mean of 5 samples for the analysis.

The probabilities of weekly Google searches for “Elf Bar” and “JUUL,” from January 1, 2022, to December 31, 2023, were plotted to visualize the temporal trends. We calculated the percentage changes in Elf Bar after JUUL’s suspension in July 2022 for each state using the following formula:



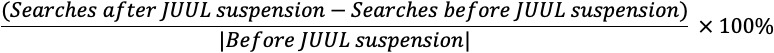



Weekly probabilities of the US public interest in Elf Bar and JUUL were calculated Using Joinpoint Regression Analysis Software (version 5.0.2; National Cancer Institute, May 2023). This joinpoint regression software uses regression modeling to assess trends while searching for temporal trend changes at time points known as joinpoints and estimating the regression function from previous joinpoints [35]. The number of joinpoints is calculated using a weighted Bayesian information criterion through the data-dependent selection method [36], and the analysis criteria were set to detect up to 5 joinpoints. This joinpoint regression analysis technique can capture nonlinear inflection points and trends in the data and provide a more nuanced understanding of the dynamics of information-seeking behavior on the internet. Weekly percentage changes between trend-change points were determined using 95% CIs.

## Results

### Overview

**Figure 1:**
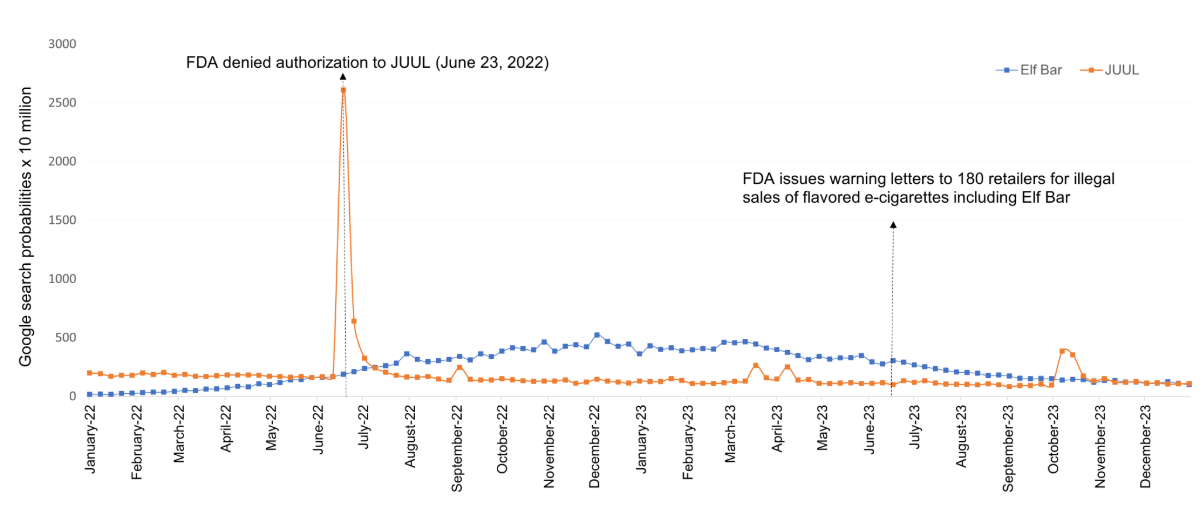
Weekly Google searches comparing “Elf Bar” and “JUUL” in 2022-2023 (105 weeks). FDA: Food and Drug Administration.

Figure 1 shows that the search interest in the Elf Bar was lower (highest weekly probability: 17.3 × 10^7^) than interest in the “JUUL” (highest weekly probability: 198.4 × 10^7^) at the beginning of 2022. Starting from week 24 (June 12, 2022), Elf Bar search interest (169.1 × 10^7^) exceeded “JUUL” (165.2 × 10^7^) and continued to steadily increase. During week 25, the search probability of JUUL peaked at 2609.3 × 10^7^. During that week (June 23, 2022), the US FDA denied JUUL marketing authority in the United States (temporarily suspending JUUL products from the US market). After this time, online interest in JUUL decreased, reaching an all-time low of 83.9 × 10^7^ on September 3, 2023 (week 88). Alternatively, Elf Bar search interest surpassed that of JUUL, again, on week 28 (July 10, 2022) and steadily increased over time, with the highest weekly probability of 523.2 × 10^7^ reported on week 49 (December 4, 2022).

### Elf Bar Searches at the State Level

**Figure 2:**
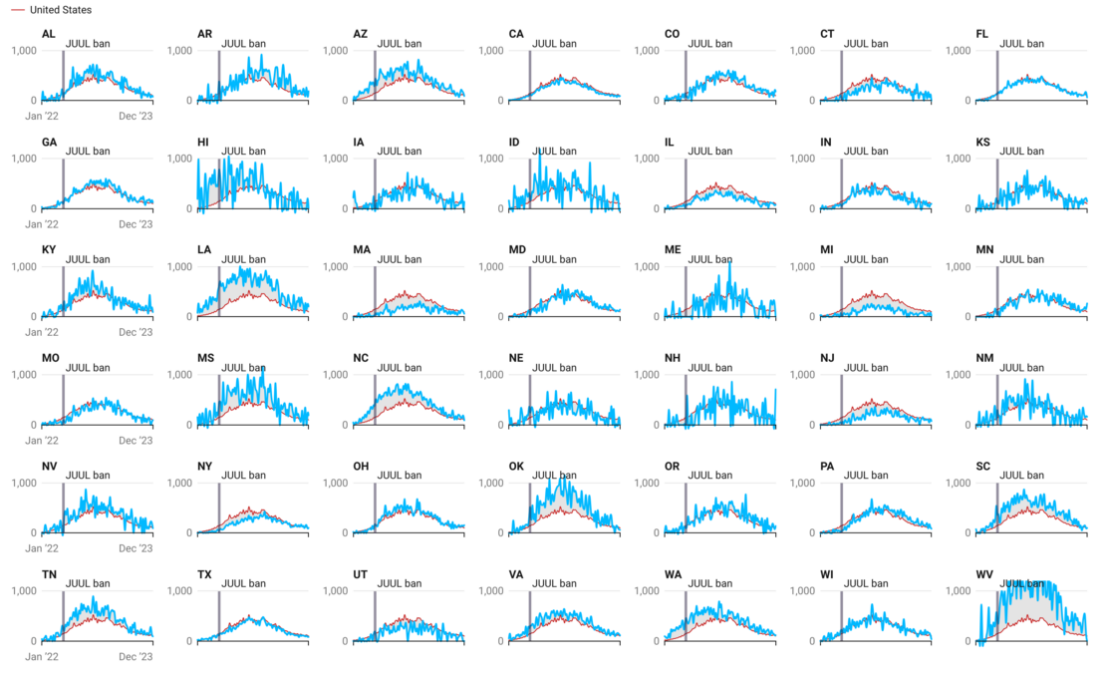
“Elf Bar” weekly search volume from January 1, 2022, to December 31, 2023. The API suppressed data and excluded 8 states (Alaska, Delaware, Montana, North Dakota, Rhode Island, South Dakota, Vermont, and Wyoming) and the District of Columbia with small populations and data uncertainty. API: application programming interface.

**Figure 3:**
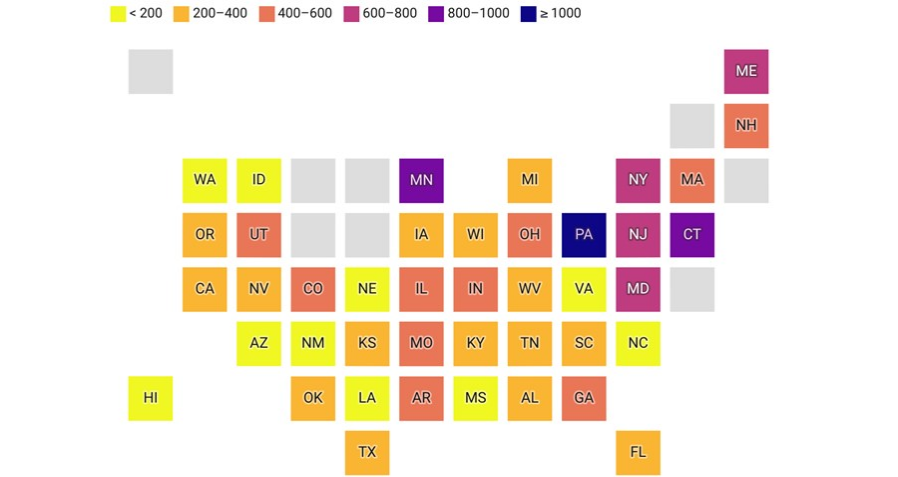
Percentage change in mean search volumes of Elf Bar after JUUL suspension in June 2022.

Trends in monthly Elf Bar search volumes for 42 states from January 1, 2022, to December 31, 2023, are shown in Figure 2. Overall, there was an upward trend in Elf Bar–related search volumes across all states after the JUUL suspension. West Virginia (866.4 × 10^7^), Louisiana (579.2 × 10^7^), Ohio (542.8 × 10^7^), Mississippi (504.2 × 10^7^), and Pennsylvania (480.6 × 10^7^) had the highest mean search volume for Elf Bar, whereas Michigan (118.2 × 10^7^), Massachusetts (144.5 × 10^7^), New Hampshire (167.2 × 10^7^), Texas (196.5 × 10^7^), and Illinois (198.1 × 10^7^) had the lowest. However, after the JUUL suspension in July 2022, changes in the percentages in Elf Bar searches were highest in Pennsylvania (1010%), Minnesota (872.5%), Connecticut (803.5%), New York (738.1%), and New Jersey (702.9%), whereas states such as Hawaii (–13.1%), Virginia (76.5%), Nebraska (76.6%), Louisiana (80.2%), and Idaho (95.2%) recorded the lowest search interest (see Figure 3).

### Trends in Elf Bar and JUUL Searches

**Figure 4:**
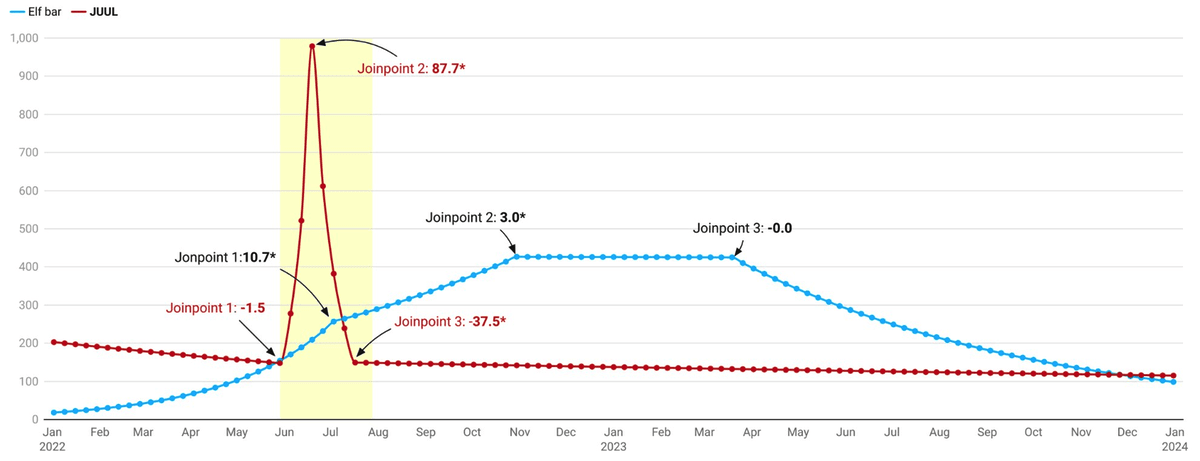
Joinpoint analysis of the weekly search trend of Elf Bar and JUUL from January 1, 2022, to December 31, 2023. *Significant at *P*<.05. Three joinpoints identified.

From January 1, 2022, to December 31, 2023, three joinpoints for Elf Bar and JUUL were identified, indicating 3 distinct trends or changes in weekly searches that all demonstrated a significant search interest during the study period (Figure 4). Overall, there was an average increase of 1.6% (95% CI 1.5-1.7; *P*<.001) in Elf Bar’s weekly search probability and an average decrease of –0.5% (95% CI –0.8 to –0.2; *P*<.001) in JUUL’s weekly search probability (Table 1).

**Table 1:** Joinpoint analysis of the weekly search trend of Elf Bar and JUUL.

Search term and period^a^			Weekly percentage change (%; 95% CI)		*P* value
**Elf Bar**					
	January 1 to July 3, 2022	10.7 (10.2 to 11.3)		<.001	
	July 3 to October 30, 2022	3.0 (2.1 to 4.6)		<.001	
	October 30 to March 19, 2023	–0.0 (–1.1 to 0.8)		.97	
	March 19 to December 31, 2022	–3.5 (–3.8 to –3.3)		.001	
	January 1, 2022, to December 31, 2023^b^	1.6 (1.5 to 1.7)		<.001	
**JUUL**					
	January 1 to May 29, 2022	–1.5 (–3.9 to 0.3)		.09	
	May 29 to June 19, 2022	87.7 (35.9 to 123.9)		.001	
	June 19 to July 17, 2022	–37.5 (–53.1 to –23.8)		.001	
	July 17 to December 2023	–0.3 (–0.6 to –0.02)		.03	
	January 1 to December 31, 2023^b^	–0.5 (–0.8 to –0.2)		<.001	

^a^Three joinpoints.

^b^Average weekly percentage change during the study period.

Briefly, before JUUL’s marketing denial order on June 23, 2022, the search interest for Elf Bar increased by 10.7% (95% CI 10.2%-11.3%; *P*<.001) per week from January to July 3, 2022. After July, the search probability for Elf Bar increased by 3.0% (95% CI 2.1%-4.6%; *P*<.001) each week until October 30, 2022, after which a negative trend was observed until December 31, 2023. In comparison, JUUL had 4 distinct time trends (1 increasing and 3 decreasing) in search interest during the study period. There was an exponential increase of 87.7% (95% CI 35.9%-123.9%; *P*=.001) in weekly search interest of JUUL during the time when the US FDA denied JUUL marketing authority in the United States, after which a significant continuous negative trend was observed.

## Discussion

In this study, we investigated the trends in Elf Bar’s and JUUL’s online searches in the United States from January 2022 to December 2023. We found that Elf Bar searches increased both before and after the FDA denied JUUL marketing authority, until October 30, 2022, where online interest in Elf Bar began to steadily decrease. These findings demonstrate that there was a decreasing online interest in JUUL and an increasing online interest in Elf Bar through 2022, which is consistent with Twitter trend data from 2021 [18]. We found there to be a peak in online interest about JUUL via GHT-API during the time that the FDA announced the suspension of JUUL products. Although this suspension was appealed by JUUL Labs the following day [37], such an announcement generated online discourse about JUUL. Including JUUL provides a comparison to understand the growing online popularity of Elf Bar, similar to prior comparisons between JUUL and Puff Bar [10]. This trend of increased interest in Elf Bar compared with JUUL continued until June 2023, around the time when the FDA announced that they had issued more than 180 warning letters to retailers for illegally selling Elf Bar products that had not received premarket approval.

Although the popularity of JUUL is well documented [5,38-42] and research suggests that the popularity of flavored disposable e-cigarette devices (ie, Puff Bar) emerged soon after the FDA’s flavor restriction for cartridge-based e-cigarette systems went into effect [10], prior research has not examined the growing online interest in the emerging e-cigarette brand Elf Bar in the United States. Our findings show that in 2022, Elf Bar searches exceeded those of JUUL, a product that controlled the e-cigarette market from 2015 to 2019 [5]. Although Puff Bar was the first disposable device to replace JUUL’s online popularity [10], emerging research suggests that Elf Bar was reported as the preferred product among youth in the United Kingdom in 2022 for reasons including better flavor and taste [17]. Further, findings from the US National Youth Tobacco Survey reported Elf Bar to be the most popular e-cigarette brand among middle school and high school students [16]. Although we cannot determine the reasons for using this search term, we can add to a growing body of literature that there was an online interest in Elf Bar during a time when it was popular among young audiences. Furthermore, research suggests that self-proclaimed small business owners are selling e-cigarettes illegally to youth on social media [43,44]. Thus, given a recent call for better regulation of tobacco e-commerce laws [45], our findings suggest the need to better understand how Google search terms were used to inform the online surveillance of e-cigarette products.

In 2019, eight states (Massachusetts, Michigan, Montana, New York, Oregon, Rhode Island, Utah, and Washington) temporarily restricted the sales of flavored e-cigarettes, in response to e-cigarette and vaping-related lung injury [46]. Since that time, 5 states (Massachusetts, New Jersey, New York, Rhode Island, and California) have prohibited the sale of all flavored e-cigarettes, and 2 states (Maryland and Utah) have restricted the sale of some flavored e-cigarettes. We found that several of these states had low weekly searches for Elf Bar after the announcement that JUUL was ordered to end sales; however, Massachusetts and New York saw a notable change in the percentage of searchers for Elf Bar after this announcement. Thus, the availability of these products may generate or curb online interest in these products. With a changing legal landscape of tobacco product (including e-cigarettes) regulation at the local, state, and federal levels [47-49], there remains a need to understand how policy changes may impact online interest in emerging tobacco products. As described in Twitter discussions, e-cigarette users often report confusion about the difference between leading products such as JUUL and Puff Bar, and some reported switching to other products rather than quitting e-cigarettes after the cartilage-based flavor restriction went into effect [23]. Thus, as state and federal tobacco regulatory policies change, GHT could help explore changes in online interest that may occur after these actions.

This study was subject to limitations. Given the limited searches provided via GT, search terms used may not capture the full landscape of interest in these brands. Further, although this study focused on the growing popularity of Elf Bar compared with JUUL, there may be other disposable devices that are also growing in popularity online. Although we were able to break down interest by location (state), using time-stamped data, we are unable to identify the demographic data of the persons looking for information about the search term. Thus, there is no way to know if the audience searching for Elf Bar was legally old enough to purchase the product (aged 21 years and older) or was younger than this age. Understanding online customers’ age could help to understand policy enforcement of the local, state, and federal Tobacco 21 laws. We also used weekly GHT data rather than data captured monthly, which could provide a more detailed response to the FDA’s JUUL suspension. Further, Google searches do not explain why the search was made; thus, we cannot confirm the reason for which people are conducting these searches. Finally, the data collected via GHT-API only capture searches for these terms online and do not capture data about use or attempts to purchase these products.

We found there to be an increased interest in Elf Bar during 2022. Although interest in JUUL increased during the time frame when the FDA announced that they were suspending the sale of JUUL products (only to be appealed the following day), the growing online interest in Elf Bar surpassed that of JUUL. Coupled with sales data and survey data about product preferences, understanding trends in online interest for particular products and brands can help inform marketing campaigns and prevention strategies that seek to reduce use among youths and young adults. Such trends data can help to describe online search history around time frames, such as announcements of tobacco regulatory responses.

## Abbreviations

COV: coefficient of variance
FDA: Food and Drug Administration
GHT-API: Google Health Trends Application Programming Interface
GT: Google Trends

## Data Availability

Data will be made available upon request.
